# Multi-Component Joint Maintenance Decision for Electro-Hydraulic Servo Fatigue Testing Machine Based on Multi-Head Deep Reinforcement Learning

**DOI:** 10.3390/s26134087

**Published:** 2026-06-27

**Authors:** Peng Liu, Guotai Huang, Jialu Xi, Jiaqi Wu

**Affiliations:** School of Mechanical and Aerospace Engineering, Jilin University, Changchun 130022, China

**Keywords:** electro-hydraulic servo fatigue testing machine, multi-component joint maintenance, deep reinforcement learning, hybrid-state Markov decision process, opportunistic maintenance

## Abstract

To address the challenge of maintenance decision-making for critical components in electro-hydraulic servo material fatigue testing machine, characterized by weak state observability and difficulty in degradation prediction, a multi-component joint maintenance decision-making method based on multi-head deep reinforcement learning is proposed. Considering the heterogeneity of the degradation mechanisms and observation methods for the four components—bearing beam, fixture, main machine sensors, and hydraulic oil tank—a continuous-discrete hybrid state Markov decision process (HS-MDP) is constructed. To account for differences in maintenance strategies across components, a differentiated discrete action space for each component is designed, and engineering feasibility constraints are explicitly integrated into the policy through action masking. A data-quality loss term, determined by the degradation level of the sensors, is introduced into the reward function to align the optimization objective with the metrological properties of the fatigue testing machine. Based on the Branching Dueling DQN framework, a Q-network structure is constructed, incorporating a shared encoder, an inter-component attention mechanism, and multi-head branched outputs. Taking a 100 kN electro-hydraulic servo fatigue testing machine as a case study, comparisons with baseline strategies such as periodic maintenance, threshold-based condition-based maintenance (CBM), independent DQN, and PPO indicate that the proposed method reduces the average annual total cost by 60.3% compared to periodic maintenance and by 42.6% compared to threshold-based CBM. The number of failures decreases from 9.8 times/year to 1.4 times/year, while data efficiency increases from 82.1% to 96.2%. Ablation experiments and robustness tests further verify the critical contributions of three key design elements: action masking, inter-component attention, and data-quality loss.

## 1. Introduction

Intelligent operation and maintenance of electro-hydraulic servo fatigue testing equipment is pivotal for ensuring the continuity of material performance evaluation tasks. The coexistence of heterogeneous degradation characteristics across the internal load-bearing structures, actuators, and measurement-and-control units makes traditional single-component maintenance models struggle to balance high reliability with economic efficiency. Coordinated maintenance of multi-component systems involves not only the independent physical degradation processes of each component, but it is also strongly influenced by the “opportunistic maintenance” effect induced by economic interdependencies among components.

This section first reviews the evolution of multi-component degradation modeling theory, examining the use of continuous stochastic processes and discrete state-transition models to describe heterogeneous failure mechanisms in complex mechanical systems. It then focuses on recent advances in deep reinforcement learning (DRL) for handling large-scale action spaces, embedding engineering logic constraints, and optimizing multi-objective cost functions. Finally, drawing on architectures such as branching networks and attention mechanisms, this section provides the theoretical underpinning for the joint maintenance decision framework proposed in this study, which is based on a Hybrid-State MDP (HS-MDP) and a multi-head branching Q-network (BDQ).

1. Degradation modeling of complex systems and multi-component maintenance logic.
  Degradation modeling of multi-component systems is the physical foundation for maintenance decision-making. In the maintenance management domain, de Jonge et al. conducted a systematic review of maintenance optimization models, emphasizing the impact of modeling accuracy on the effectiveness of decisions [1]. Nicolai et al. further elucidated the importance of economic interdependencies in multi-component systems, providing an early theoretical framework for “opportunistic maintenance” [2]. For structural components in fatigue testing equipment—such as bearing beams and fixture—that exhibit continuous cumulative damage, Thomas noted that maintenance of multi-unit systems should account for stochastic evolution processes [3]. Saha et al. systematically surveyed the application of Wiener processes in degradation prediction for highly reliable products [4], while Pan et al. explored multi-performance-index reliability modeling based on Gamma processes [5].
  However, the states of sensors and the hydraulic oil system in testing machines often exhibit discrete jumps as revealed by periodic calibration or laboratory analysis. Keizer et al. effectively modeled discrete degradation states using a partially observable Markov decision process (POMDP) [6]. In dealing with such complex systems, Si et al. reviewed data-driven techniques for remaining useful life (RUL) prediction [7]. Grall et al. pointed out that optimal maintenance policies must balance the benefit of a single repair against the fixed cost of a system-wide shutdown [8]. Castanier et al. further explored combining continuous degradation dynamics with discrete decision logic, laying the mathematical groundwork for continuous–discrete hybrid-state modeling [9].
2. Deep reinforcement learning for maintenance decision optimization.
  In the face of the exponential growth of the state space in multi-component systems, deep reinforcement learning (DRL) offers a model-free pathway for decision optimization in high-dimensional environments [10]. Zhang and Si were among the first to introduce DRL into condition-based maintenance (CBM) planning for multi-component systems, achieving an end-to-end mapping from multi-source monitoring signals to optimal maintenance actions [11]. To capture complex inter-component interactions, Do et al. and Chen et al. explored structure-dependent multi-component maintenance policies, demonstrating DRL’s strong capability in handling economic interdependencies [12,13]. However, as precision metrological equipment, fatigue testing machines face a unique challenge: sensor degradation can render test data out of tolerance, which are thus scrapped. This distinctive “data-quality loss” is rarely represented in general maintenance studies, making algorithms difficult to directly adapt to the metrological attributes of fatigue testing equipment.
3. Action-space decoupling, engineering masks, and coordination mechanisms.
  When maintenance decisions involve multiple heterogeneous components, the size of the joint action space grows exponentially. To address this challenge, the Dueling DQN architecture proposed by Wang et al. decouples the state value and action advantage [14]; building on this, the branching multi-head architecture (Branching BDQ) proposed by Tavakoli et al. greatly reduces sample complexity in high-dimensional discrete-action environments by decoupling the output heads [15]. Meanwhile, real maintenance operations must adhere to strict engineering constraints. De Giacomo et al. articulated the key role of action masking in ensuring logical compliance in reinforcement learning [16], and Huang et al. demonstrated that explicitly injecting state masks can significantly shrink the search space and accelerate policy convergence [17].
  In addition, modeling inter-component interactions has become crucial for improving collaborative maintenance efficiency. With the widespread adoption of attention mechanisms in feature engineering, researchers have introduced them into the PHM field to capture cross-component degradation synergies [18]. For example, Bai et al. combined attention mechanisms with DRL and improved maintenance decision accuracy by adaptively fusing multi-scale information [19]. Studies by Vu et al. and Olde Keizer et al. also show that modeling economic synergies among components can significantly enhance overall system availability [20,21]. These advanced algorithmic architectures provide the core tools in this study for injecting action masks and enabling cross-component coordinated decision-making in complex maintenance scenarios of fatigue testing equipment.

In summary, substantial progress has been made in multi-component maintenance modeling and reinforcement learning algorithm optimization. Nevertheless, existing research mostly focuses on homogeneous-component systems or simplified single-degradation models and lacks in-depth analysis of fatigue testing equipment, which integrates continuous degradation of precision mechanical structures with discrete deterioration of measurement-and-control systems—i.e., a continuous–discrete hybrid-state system. Consequently, for test machines exhibiting multi-source heterogeneous degradation, how to achieve intelligent decision-making that also accounts for metrological data quality under the dual constraints of an exploding action space and engineering feasibility remains largely unexplored. There is an urgent need to develop a multi-head branching deep reinforcement learning architecture that can explicitly handle action-masking constraints and inter-component attention dependencies.

Contributions: It should be stated at the outset that the algorithmic primitives employed in this work—the branching value decomposition, the dueling architecture, the scaled dot-product attention, and logit-level action masking—are adopted from the literature rather than newly proposed. The novelty of this study lies instead in the problem it formalizes, in one domain-specific modeling element, and in an integration whose components are shown to be individually necessary:

(1) A maintenance-decision formulation for a previously underexplored equipment class. Unlike production machinery, an electro-hydraulic servo fatigue testing machine is itself a metrological instrument: its load-bearing structures degrade continuously, whereas its measurement-and-control units degrade in discrete, calibration-revealed jumps. We cast the joint maintenance of these heterogeneous subsystems as a continuous–discrete hybrid-state MDP (HS-MDP), in which Wiener processes and operating-condition-modulated Markov chains are coupled through a shared condition variable. To the best of our knowledge, the coupling of continuous precision-mechanical degradation with discrete measurement-system degradation has not previously been formalized as a single maintenance-decision problem.
(2) A data-quality loss term that aligns the policy with the metrological function of the equipment. Because the deliverable of a testing machine is valid measurement data, sensor degradation silently drives test results out of tolerance and causes data to be scrapped. We introduce a data-quality cost $c_{\mathrm{qual}\;}\left(z_{t}^{\left(3\right)}\right)$ that increases stepwise with the sensor degradation level—an objective dimension largely absent from general maintenance studies. Its effect on the learned policy is isolated in the ablation study, where it is shown to reshape sensor-maintenance behavior rather than merely re-label an existing cost.
(3) A purpose-built integration whose elements are empirically necessary. The hard/soft engineering action masks, the inter-component attention for opportunistic coordination, and the multi-head dueling heads are combined so that each component decision is simultaneously feasibility-constrained, condition-aware, and aware of the other components’ states. The contribution of each element, and the physical interpretability of the learned attention weights, are examined in the ablation and attention analyses.

## 2. Problem Modeling and Hybrid-State Markov Decision Process

### 2.1. Maintenance Decision Scenario

During the long-term operation of an electro-hydraulic servo material fatigue testing machine, four critical components—the bearing beam, fixture, main force/displacement sensor, and hydraulic oil tank—undergo degradation processes driven by different mechanisms: The bearing beam experiences a gradual deviation in horizontality under prolonged eccentric and high-frequency alternating loads, exhibiting a continuous accumulation characteristic. The fixture undergoes contact wear due to repeated clamping and loading, with the wear amount similarly exhibiting continuous accumulation. The zero drift and sensitivity of the main sensors degrade over time and manifest as discrete levels observable through periodic calibration. The contamination level of hydraulic oil, following standards such as NAS 1638, intrinsically falls into discrete levels. At each decision time step t, the maintenance decision agent outputs a joint action $a_{t}\;=\;({a_{t}}^{1},\;{a_{t}}^{2},\;{a_{t}}^{3},\;{a_{t}}^{4})$, where $a_{t}^{i}$ represents the action chosen for the i-th component. After executing the action, the system transitions to a new state $s_{t+1}$ and incurs an immediate cost $c_{t}$. The objective of the decision-making process is to find a policy π:S→A that minimizes the expected long-term discounted cumulative cost:

(1) $$\pi^{*}=arg\underset{\pi }{min}\;E_{\pi }\left[\sum_{t=0}^{T}\;\gamma^{t}c_{t}\right]$$

where γ∈(0,1) is the discount factor, and T represents the decision time horizon.

### 2.2. Hybrid-State Markov Decision Process

Considering the heterogeneity in the degradation mechanisms and observation methods of the four components, this paper adopts the Hybrid-State Markov Decision Process (HS-MDP) for modeling. HS-MDP is defined as a quintuple (*S*, *A*, *P*, *R*, *γ*), where the state space is the Cartesian product of the continuous subspace and the discrete subspace:

(2) $$S=X\times Z\subseteq R^{d_{c}}\times \prod_{j}\;\left\{1,\ldots ,K_{j}\right\}$$

**Figure 1:**
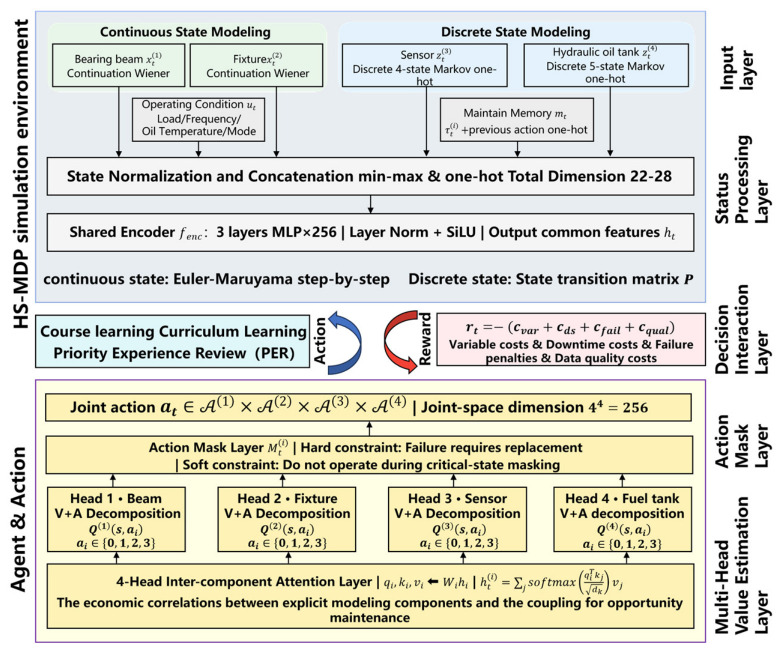
Maintenance Decision-Making Framework Based on Multi-Head Deep Reinforcement Learning.

The subspace X accommodates the degradation variables of the bearing beam and fixture, while the discrete subspace Z includes the degradation levels of the sensors and hydraulic oil tank. The action space A is the Cartesian product of the action subspaces of the four components. The transition function $P(s_{t+1}|s_{t},a_{t})$ propagates the continuous components using stochastic differential equations and the discrete components based on a state transition matrix, with both coupled through the shared operating condition variable $u_{t}$. The reward function $R(s_{t},a_{t})$ integrates maintenance costs, downtime costs, failure costs, and data-quality loss. This modeling approach not only ensures mathematical rigor but also considers the engineering relevance of the physical degradation characteristics of different components. The overall technical framework is illustrated in Figure 1.

## 3. Design of Hybrid State Space

### 3.1. Overall Structure of the State Vector

Define the system state vector at time t as:

(3) $$s_{t}=\left[x_{t}^{\left(1\right)},x_{t}^{\left(2\right)},z_{t}^{\left(3\right)},z_{t}^{\left(4\right)},u_{t},m_{t}\right]$$

where $x_{t}^{1}$ and $x_{t}^{2}$ are the continuous state sub-vectors for the bearing beam and fixture, respectively; $z_{t}^{3}$ and $z_{t}^{4}$ are the discrete state one-hot encodings for the main machine sensors and hydraulic oil tank, respectively; $u_{t}$ is the global operating condition vector; and $m_{t}$ is the maintenance memory vector.

### 3.2. Continuous State Modeling: Bearing Beam and Fixture

For the bearing beam, the core degradation variable is the horizontality deviation *hₜ*. Define the composite horizontality deviation as $h_{t}=\sqrt{{\left(h_{t}^{x}\right)}^{2}+{\left(h_{t}^{y}\right)}^{2}}$, and model it using a drift–diffusion Wiener process:

(4) $$dh_{t}=\mu_{h}\left(u_{t},e_{t}\right)dt+\sigma_{h}dB_{t}$$

where *Bₜ* is a standard Brownian motion and σₕ is the diffusion coefficient. The drift term adopts a multiplicative structure to reflect the coupling between operating conditions and degradation:

(5) $$\mu_{h}\left(u_{t},e_{t}\right)=\mu_{h,0}\cdot {\left(\frac{L_{t}}{L_{0}}\right)}^{p_{1}}\cdot {\left(\frac{f_{t}}{f_{0}}\right)}^{p_{2}}\cdot \left(1+\lambda e_{t}\right)$$

where *Lₜ* and *fₜ* are the current load amplitude and loading frequency, *eₜ* is the eccentric loading coefficient, and *p*_1_, *p*_2_ and λ are coupling parameters. This structure enables the policy to learn the decision logic of preemptive leveling under heavy-load, high-frequency, and eccentric loading conditions. The continuous state sub-vector for the bearing beam is defined as ${x_{t}}^{1}\;=\;(h_{t},\;{ḣ}_{t},\;{D_{t}}^{1},\;e_{t})\;\in \;R^{4}$, where *ḣₜ* is the drift-rate estimate over a recent window and Dt1 = ∫0t (Lτ/L0)^p1 dτ is the cumulative load equivalent. The failure criterion is $h_{t}\;\geq \;h\_crit$.

For the fixture, the degradation variable is the contact wear *wₜ*, which is likewise modeled using a Wiener process:

(6) $$dw_{t}=\mu_{w}\left(u_{t}\right)dt+\sigma_{w}dB_{t}$$

where $B_{t}$ is a standard Brownian motion, the drift coefficient $\mu_{w}\left(u_{t}\right)$ is related to the load and the number of clamping operations, and $\sigma_{w}$ is the diffusion coefficient. The continuous state sub-vector is defined as *xₜ*^2^ = (*wₜ*, *ẇₜ*, *nₜ*^2^) ∈ ℝ^3^, where *nₜ*^2^ is the cumulative number of clamping operations (log-normalized). The failure criterion is $w_{t}\geq w_{\mathrm{crit}\;}$.

The drift–diffusion Wiener process is adopted for the bearing beam and the fixture for three reasons. First, both the horizontality deviation and the contact wear are cumulative quantities whose one-step increment is governed by the current load condition rather than the full degradation history, so an independent-increment (Markov) model is appropriate. Second, the additive diffusion term represents the measurement noise of the horizontality/wear sensing together with the short-term stochasticity of the load profile, while the operating-condition-dependent drift (Equation (5)) lets the mean degradation rate respond to load amplitude, loading frequency, and eccentricity. Third, the Wiener process admits a closed-form first-passage-time (inverse-Gaussian) distribution to the failure threshold, which is convenient for reliability evaluation. Over the service horizon considered here, the drift dominates the diffusion, so the simulated trajectories are effectively monotone. Strictly monotone alternatives such as the Gamma or inverse-Gaussian process could be substituted without altering the HS-MDP or the decision algorithm.

In the simulation, the two continuous states are advanced in discrete time using the Euler–Maruyama scheme.

### 3.3. Discrete State Modeling: Sensors and Hydraulic Oil Tank

A discrete-state Markov chain is used for the sensors and the oil tank because both degradation variables are intrinsically ordinal and are observed only at discrete inspection epochs: sensor health is graded into levels by periodic calibration of zero drift and sensitivity, and oil cleanliness is graded into NAS 1638 contamination classes. A continuous state would therefore be neither observable nor physically meaningful for these two subsystems. The memoryless assumption—that the probability of advancing to the next level depends only on the current level and the prevailing operating condition—is consistent with the level-to-level structure of the calibration and oil-analysis record. Degradation is taken to be monotone in the absence of maintenance, which yields the upper-triangular transition matrices with an absorbing failure state; the dependence on operating conditions is captured through a stress-acceleration factor on loading frequency for the sensors (Equation (7)) and an Arrhenius-type factor on oil temperature for the oil tank (Equation (8)).

The degradation of the main machine sensors primarily manifests as zero drift and sensitivity loss, which are detected through periodic calibration. A four-level discrete state is adopted for the sensors, with *K*_3_ = 4 and ${z_{t}}^{3}\;\in \;\{1,\;2,\;3,\;4\}$. The hydraulic oil quality in the oil tank is classified according to NAS 1638 contamination levels, and a five-level discrete degradation state is used, with $K_{4}=5$ and $z_{t}^{\left(4\right)}\in \left\{1,2,3,4,5\right\}$. The meanings of each level are provided in Table 1.

Sensor degradation follows a Markov chain, with the state transition matrix P3(uₜ) ∈ R^(4×4) taking an upper-triangular form (degradation is monotonic and irreversible in the absence of maintenance), and state 4 is absorbing. The dependence on operating conditions is reflected by making the transition probabilities depend on the loading frequency:

(7) $$p_{i,i+1}^{\left(3\right)}\left(u_{t}\right)=p_{i,i+1}^{\left(3\right),0}\cdot \exp\left(\beta \cdot f_{t}\right)$$

The hydraulic oil tank state transition matrix $P^{4}(\theta ₜ)$ likewise has an upper-triangular structure and depends on the oil temperature θₜ:

(8) $$p_{i,i+1}^{\left(4\right)}\left(\theta_{t}\right)=p_{i,i+1}^{\left(4\right),0}\cdot \exp\left(\alpha \left(\theta_{t}-\theta_{0}\right)\right)$$

The higher the oil temperature, the faster the oil oxidizes, and the transition probabilities increase. The discrete states are one-hot encoded when fed into the network: $onehot({z_{t}}^{3})\;\in \;{\left\{0,1\right\}}^{4}$, $onehot({z_{t}}^{4})\;\in \;{\left\{0,1\right\}}^{5}$.

### 3.4. Global Operating Conditions and Maintenance Memory

Operating conditions have a significant impact on the degradation rates of each component and must be included in the state: $u_{t}=\left[L_{t},f_{t},\theta_{t}^{\mathrm{env}\;},{Mode}_{t}\right]$, where *Lₜ* is the normalized load amplitude level, $\theta_{t}^{\mathrm{env}}$ is the ambient temperature, and Modeₜ is a one-hot encoding of the test mode (e.g., tensile fatigue, bending fatigue, load-holding). Maintenance memory vector $m_{t}=\left[\tau_{t}^{\left(1\right)},\tau_{t}^{\left(2\right)},\tau_{t}^{\left(3\right)},\tau_{t}^{\left(4\right)},{\;\mathrm{LastAct}}_{t}\right]$, where $\tau_{t}^{i}$ denotes the operating time since the last maintenance of component *i*, used to distinguish between “a newly replaced component” and “an old component whose degradation happens to be similar.” After min–max normalization of continuous components and one-hot encoding of discrete components, they are concatenated to form the complete state vector.

## 4. Multi-Head Joint Action Space and Reward Function

### 4.1. Component-Differentiated Action Space

An exclusive action set is designed for each component based on its physical maintenance measures, uniformly covering a four-tier maintenance hierarchy: no action—inspection/prevention—minor repair—replacement. At time *t*, the agent outputs a joint action $a_{t}\;=\;({a_{t}}^{1},\;{a_{t}}^{2},\;{a_{t}}^{3},\;{a_{t}}^{4})\;\in \;A_{1}\;\times \;A_{2}\;\times \;A_{3}\;\times \;A_{4}$, and the joint action space has a cardinality of $4^{4}\;=\;256$. Through multi-head decomposition, each output head makes decisions only within a size-4 action space, significantly reducing sample complexity. The action designs for each component are shown in Table 2.

For the bearing beam and fixture, moderate maintenance is modeled as imperfect repair, with restoration ratios $\rho_{1}$ and $\rho_{2}\;\sim \;Beta(\alpha ,\;\beta )$, and an expected restoration rate E[ρ] ∈ [0.7, 0.9]. This modeling reflects the diminishing returns under the “leveling → re-degradation → re-leveling” sequence, forcing the policy to balance over the long term between repeated low-cost leveling and a one-time high-cost replacement—a dilemma typical near the end of the bearing beam’s service life in practice. The differentiated rollback magnitudes in the oil tank actions compel the policy to learn hierarchical decision-making rather than uniformly selecting the strongest maintenance action.

### 4.2. Action Feasibility Constraints and Action Masking

Not all actions are meaningful in every state. This study explicitly injects engineering priors into the policy via an action mask $M_{t}^{\left(i\right)}\in \{0,1{\}}^{|A^{\left(i\right)}|}$. Under hard rules, a component is forced to be replaced upon failure (e.g., $h_{t}\geq h_{\mathrm{crit}\;}\Rightarrow M_{t}^{\left(1\right)}=\left[0,0,0,1\right]$). Under soft constraints, when the horizontality deviation approaches the tolerance limit, the “no action” option is masked ($h_{t}\geq 0.8h_{\mathrm{crit}\;}\Rightarrow M_{t}^{\left(1\right)}=\left[0,1,1,1\right]$) to avoid missing critical maintenance windows. Invalid actions (e.g., when ${z_{t}}^{3}=1$, calibration is meaningless) can likewise be masked. The mask is implemented at the network output layer by setting the corresponding logits to −∞:

(9) $$\tilde{Q}\left(s_{t},a^{\left(i\right)}\right)=Q\left(s_{t},a^{\left(i\right)}\right)+logM_{t}^{\left(i\right)}\left(a^{\left(i\right)}\right)$$

This hard-constraint approach is more stable and more sample-efficient than reward-penalty shaping. Ablation experiments indicate that it is the design element contributing the most to the final performance.

### 4.3. Joint Execution and Multi-Objective Reward Function

To appropriately account for downtime costs, this study defines a joint execution rule: if any aₜⁱ > 0, a downtime event is triggered. The fixed downtime cost is charged once per event, $c_{ds}\left(a_{t}\right)=C_{\mathrm{setup}\;}\cdot 1\left[\exists i:a_{t}^{\left(i\right)}>0\right]$—this is the core manifestation of the opportunistic maintenance effect, i.e., performing maintenance actions on multiple components during a single shutdown does not increase the fixed downtime cost. The downtime duration follows parallel execution and is taken as the longest action duration, $T_{down}\left(a_{t}\right)=\underset{i}{max}\;\tau^{\left(i\right)}\left(a_{t}^{\left(i\right)}\right)$. The total variable downtime cost equals the sum of materials and labor costs of the actions, plus the production loss rate per unit downtime multiplied by the downtime duration.

The reward function converts the maintenance objectives into a scalar signal:

(10) $$r_{t}=-\left[c_{\mathrm{var}\;}\left(a_{t}\right)+c_{\mathrm{ds}\;}\left(a_{t}\right)+c_{\mathrm{fail}\;}\left(s_{t}\right)+c_{\mathrm{qual}\;}\left(z_{t}^{\left(3\right)}\right)\right]$$

where $c_{\mathrm{var}\;}\left(a_{t}\right)$ is the sum of variable costs for all proactive maintenance operations, and $c_{\mathrm{ds}\;}\left(a_{t}\right)$ is the fixed downtime cost. The failure penalty is $c_{\mathrm{fail}\;}\left(s_{t}\right)=\sum_{i}\;c_{f}^{\left(i\right)}1\left[s_{t}^{\left(i\right)}\in F^{\left(i\right)}\right]$, where $F^{\left(i\right)}$ is the failure-state set of component *i*, and $c_{f}^{\left(i\right)}$ is set far higher than the preventive replacement cost to reflect the severe consequences of failures (e.g., specimen damage, secondary damage). The term $c_{\mathrm{qual}\;}\left(z_{t}^{\left(3\right)}\right)$ denotes the test-data-quality loss and is a distinctive element of the reward function in this study; its value increases stepwise with the sensor degradation level: $c_{\mathrm{qual}\;}\left(1\right)=0$ when ${z_{t}}^{3}\;=\;1,$ and for ${z_{t}}^{3}\;=\;2,\;3,\;4$ it takes $\kappa_{1}\cdot N_{\mathrm{test}}\left(t\right)$, $\kappa_{2}\cdot N_{\mathrm{test}}(t)$, $\kappa_{3}\cdot N_{\mathrm{test}}(t)$, respectively, where $N_{\mathrm{test}}$ is the number of tests completed at the current time step and $\kappa_{1}\;<\;\kappa_{2}\;\ll \;\kappa_{3}$. This term aligns the policy with the metrological characteristics of the fatigue testing machine—an often overlooked yet crucial cost dimension in general maintenance decision studies.

## 5. Multi-Head Deep Reinforcement Learning Method

### 5.1. Algorithm Selection and Motivation

For the multi-component collaborative maintenance decision model of the electro-hydraulic servo fatigue testing machine constructed in this study, one of the core challenges lies in the high-dimensional, multi-dimensional discrete action space. The system comprises four key components, each associated with four mutually exclusive maintenance actions (no action, inspection/prevention, intermediate/partial repair, complete replacement), forming a complex joint action space of size 4^4^ = 256. When selecting the base deep reinforcement learning (DRL) algorithm, it is essential to carefully balance the structural characteristics of the action space, sample efficiency, and the difficulty of embedding engineering feasibility constraints.

State-of-the-art policy-gradient methods (e.g., TD3 and SAC) perform well in industrial control but are natively designed for continuous action spaces. Forcibly applying them to the multi-dimensional discrete maintenance setting considered in this study typically requires continuous relaxations (e.g., Gumbel-Softmax reparameterization) or hard discretization of continuous outputs. Such transformations introduce substantial gradient-estimation noise and quantization errors, markedly increasing optimization difficulty and making convergence challenging in real engineering contexts where work-order data and physical samples are costly. Even with discrete-space variants (e.g., SAC-Discrete), these methods must construct and maintain a global probability distribution over the entire joint action space. As the number of maintained components grows, the joint action space explodes exponentially (M^N), forcing traditional architectures to output and optimize a policy vector as large as 256 dimensions in this case, which leads to severe parameter redundancy, poorer sample efficiency, and degraded training stability. More critically, continuous-control algorithms or traditional global-discrete policies do not natively and cleanly support per-component action masking. Under hard engineering constraints such as “a failed component must be replaced,” imposing constraints on a 256-dimensional global joint distribution or on continuous outputs is highly complex and cannot guarantee that, during early exploration, the agent will avoid safety-violating invalid actions with 100% certainty.

Based on the above considerations, this study adopts the Branching Dueling DQN (BDQ) as the core base algorithm. Through its distinctive branching structure and dueling decomposition, the BDQ architecture exhibits the following three notable advantages tailored to multi-component collaborative maintenance scenarios:

(1) Action-space decoupling and linearized parameterization: BDQ is specifically designed for multi-discrete dimensions with independent Q-value output branches, assigning a dedicated output head to each component. This reduces the parameter complexity at the output layer of policy network from the exponential growth of the joint space (M^N = 256) to linear growth ($\sum_{i=1}^{N}\;M_{i}=4\times 4=16$), effectively eliminating action-space explosion and greatly improving sample efficiency in multi-dimensional discrete control.
(2) Native and seamless support for action masking: Because the action spaces of individual maintenance components are fully decoupled at the output, the agent can conveniently apply component-specific state masks directly on each branch. By setting the logits of actions that do not meet engineering feasibility or logical constraints to −∞ at the network output layer, the agent is guaranteed not to trigger dangerous or invalid maintenance combinations throughout the entire training process. Compared with traditional reward shaping, this hard-constraint mechanism delivers higher training stability and faster convergence.
(3) Capturing state–action advantages: BDQ integrates a dueling architecture within each branch, precisely decomposing the Q-value of each component into the global state value V(s) and the component action advantage A(s, a). In the operation of the fatigue testing machine, states such as severe degradation or critical failures occur infrequently; the dueling architecture enables the agent to accurately assess the overall safety value of system without frequently exploring every specific maintenance combination, thereby effectively mitigating learning lag under sparse major maintenance events.

### 5.2. Multi-Head Q-Network Architecture with Inter-Component Attention

The proposed network architecture consists of three parts: a shared encoder, an inter-component attention layer, and branching heads.

The shared encoder maps the state vector *sₜ* to a common feature representation: $h_{t}=f_{enc}\left(s_{t};\theta_{enc}\right)$. The function $f_{enc}$ is a three-layer multilayer perceptron with 256 neurons per layer, using LayerNorm and SiLU activation. Sharing the low-level representation improves the sample efficiency of the component-wise decisions.

The inter-component attention layer is introduced to explicitly model economic correlations among components and correlations induced by opportunistic maintenance. The shared feature *hₜ* is linearly projected into each component’s query, key, and value vectors $q_{i},k_{i},v_{i}$, and then the cross-component attention-weighted features are computed:

(11) $${\tilde{h}}_{t}^{\left(i\right)}=\sum_{j=1}^{4}\;softmax\left(\frac{q_{i}^{⊤}k_{j}}{\sqrt{d_{k}}}\right)v_{j}$$

${\tilde{h}}_{t}^{\left(i\right)}$ is the context feature for decision-making on the i-th component, integrating the state information of all four components. The engineering intuition behind this design is that when the oil tank is close to the oil-change threshold, the sensors should tend toward opportunistic calibration even if they are only mildly degraded—this cross-component dependency is precisely what the attention mechanism models.

The branching heads (with a dueling architecture) are independently configured for each component, using the dueling decomposition to split the Q-value into the state value and the advantage function:

(12) $$Q^{\left(i\right)}\left(s_{t},a^{\left(i\right)}\right)=V^{\left(i\right)}\left(s_{t}\right)+A^{\left(i\right)}\left(s_{t},a^{\left(i\right)}\right)-\frac{1}{\left|A^{\left(i\right)}\right|}\sum_{a^{\prime }}\;A^{\left(i\right)}\left(s_{t},a^{\prime }\right)$$

The Q-value of the joint action is obtained by averaging the component-wise Q-values: $Q\left(s_{t},a_{t}\right)=\frac{1}{N}\sum_{i=1}^{N}\;Q^{\left(i\right)}\left(s_{t},a_{t}^{\left(i\right)}\right)$.

### 5.3. Training Algorithm

The algorithm adopts Double DQN target construction and an experience replay mechanism. The replay buffer stores transition tuples $\left(s_{t},\;a_{t},\;r_{t},\;s_{t+1},\;M_{t+1}\right)$ (where $M_{t+1}$ is the action mask of the next state). The training objective is to minimize the temporal-difference loss:

(13) $$L\left(\theta \right)=E_{\left(s_{t},a_{t},r_{t},s_{t+1}\right)∼D}\left[{\left(y_{t}-Q\left(s_{t},a_{t};\theta \right)\right)}^{2}\right]$$

(14) $$y_{t}=r_{t}+\gamma \cdot \frac{1}{N}\sum_{i=1}^{N}\;Q^{\left(i\right)}\left(s_{t+1},\arg\underset{a^{\prime }}{max}\;{\tilde{Q}}^{\left(i\right)}\left(s_{t+1},a^{\prime };\theta \right);\theta^{-}\right)$$

where $\theta^{-}$ denotes the target network parameters, and a soft update is used: $\theta^{-}\leftarrow \tau \theta +\left(1-\tau \right)\theta^{-}$.

This study employs three training techniques: (1) reward normalization, applying online running mean/variance normalization to each cost term to prevent large terms from dominating the gradients; (2) curriculum learning, constraining the simulation horizon and reducing the stochasticity of degradation rates in early training, then gradually restoring the target difficulty once the policy has largely formed; (3) prioritized experience replay, increasing the sampling probability of transitions with larger TD errors (typically corresponding to failures or major maintenance events). Key hyperparameters are: discount factor γ = 0.99, learning rate $3\times 10^{-4}$, soft-update coefficient τ = 0.005, replay buffer capacity 10^5^, batch size 256, encoder hidden dimension 256, and number of attention heads 4.

## 6. Experimental Validation

### 6.1. Case Study and Simulation Environment

This study uses a 100 kN electro-hydraulic servo material fatigue testing machine of a certain model as the case object. The machine performs high-cycle and low-cycle fatigue tests on metal specimens, operates about 16 h per day, and runs 260 days per year. Based on the HS-MDP formalization in Section 2, Section 3, Section 4 and Section 5, a simulation environment is constructed with a time step Δt = 1 day and an episode length T = 260 steps. Degradation and cost parameters are determined jointly from the manufacturer’s maintenance manual, corporate financial records from the past 3 years, and site visits to three user organizations. The baseline horizontality drift rate of the bearing beam is $\mu_{h,0}=\;1.2\;\times \;10^{-3}\;mm/day$, the critical horizontality is h_crit = 0.50 mm, and the baseline wear rate of the fixture is $\mu_{w,0}=\;4.5\;\times \;10^{-3}\;mm/day$. The discrete transition probabilities for the sensors and the oil tank are obtained via maximum likelihood estimation from dwell times at each level in work-order data. Operating conditions *uₜ* are generated according to typical fatigue test schedules: load amplitude *Lₜ* is uniformly sampled in [0.6, 1.4], and loading frequency *fₜ* is sampled from a normal distribution within 10–30 Hz. The simulation environment is implemented using the OpenAI Gymnasium standard interface, encapsulated as a four-component joint MDP, with the software platform comprising Python 3.10, NumPy 1.26, and PyTorch 2.2.1.

The base transition probabilities of the two discrete chains are obtained by maximum-likelihood estimation from the level dwell times recorded in 3 years of calibration and maintenance work orders. For a one-step (pure-birth) chain, the dwell time in level i is geometrically distributed with parameter $p_{i,i+1}$, so the maximum-likelihood estimate is

(15) $${\hat{p}}_{i,i+1}^{0}=\frac{1}{{\bar{d}}_{i}},{\bar{d}}_{i}=\frac{1}{N_{i}}\sum_{k=1}^{N_{i}}\;d_{i}^{\left(k\right)}$$

where $d_{i}^{\left(k\right)}$ is the dwell time (in time steps) of the k-th sojourn in level i and $N_{i}$ is the number of such sojourns. These baseline estimates are referenced to the nominal operating condition $\left(f_{0},\theta_{0}\right)$; the acceleration coefficients β and α in Equations (7) and (8) are then estimated by log-linear regression of the per-condition transition rates on loading frequency and oil temperature, respectively.

It should be emphasized that the manufacturer manuals, 3-year calibration and maintenance records, financial records, and site investigations were used to identify and calibrate the parameters of the HS-MDP simulator. However, all policy training, baseline comparisons, ablation studies, robustness tests, and sensitivity analyses reported in this section were conducted offline in this data-calibrated simulation environment. No learned policy was deployed online on the physical fatigue testing machine, and no intervention decisions were executed on the real equipment during this study. Therefore, the following results should be interpreted as simulation-based evidence of decision performance rather than as direct field validation.

### 6.2. Baselines and Evaluation Metrics

To systematically evaluate the relative advantages of the proposed method (denoted BDQ-Att-Mask), five representative baselines are selected for comparison:

- S1 Periodic: Periodic maintenance policy that performs medium-intensity maintenance and scheduled replacements at fixed intervals prescribed by the manufacturer’s maintenance manual.
- S2 Threshold-CBM: Threshold-based condition-based maintenance strategy that triggers the corresponding maintenance when a component’s state exceeds a preset threshold.
- S3 Independent-DQN: Four independent DQNs (one per component) with no awareness of other components’ states, used to isolate the contribution of collaborative modeling.
- S4 PPO-MultiDisc.: PPO for multi-discrete actions, used to verify BDQ’s advantage in sample efficiency.
- S5 Vanilla-BDQ: Standard BDQ without inter-component attention or action masking, serving as the ablation baseline.

To ensure a fair comparison, Independent-DQN, PPO-MultiDiscrete, Vanilla-BDQ, and BDQ-Att-Mask were trained under the same maximum episode horizon of 260 steps and the same final environment-interaction budget of [780,000] steps. The number of executed environment steps was counted directly from the training logs rather than inferred from the number of episodes, because curriculum learning and early termination may produce episodes of different lengths. All methods used the same set of training seeds. Each seed jointly controlled network initialization, environment stochasticity, action sampling, and minibatch sampling. After training, the network parameters were frozen, and every trained policy was evaluated using the same set of 30 previously unseen evaluation seeds. The evaluation seeds were not used for hyperparameter selection.

Where algorithmically applicable, the methods shared the same state preprocessing, reward normalization, curriculum schedule, network width, discount factor, and total environment-interaction budget. Independent-DQN, Vanilla-BDQ, and BDQ-Att-Mask additionally used the same replay-buffer capacity, batch size, Double-DQN target construction, target-network update rule, exploration schedule, and prioritized-replay settings. PPO used its standard on-policy rollout and update procedure and therefore did not use an experience replay buffer.

Hyperparameters were selected using a predefined search protocol. For each DRL method, same candidate configurations were evaluated using the same tuning interaction budget and the same set of validation seeds. The configuration with the lowest mean annual total cost on the validation seeds was selected. The final 30-seed test results were obtained only after hyperparameter selection had been completed. Thus, the proposed method and all learned baselines received the same data budget and comparable hyperparameter-tuning effort.

The evaluation metrics are constructed along three dimensions: economic benefits (average annual total cost and four subcategories), reliability (average annual number of component failures), and operational availability (annual cumulative downtime and data efficiency). All metrics are reported as mean ± standard deviation over 30 independent random seeds for yearly simulations. To ensure comparability, the DRL policies’ network weights are frozen after training, and evaluation is conducted using the same seeds.

The five baselines are chosen to represent the relevant families rather than to enumerate individual papers: S1 the prevailing industrial practice (fixed schedule), S2 classical condition-based maintenance, S3 the decentralized multi-agent family (one independent learner per component), S4 the modern on-policy actor-critic family (multi-discrete PPO), and S5 the value-decomposition backbone without our additions. Recent maintenance-DRL methods—dependent-competing-risk CBM [11], structure-dependent and multi-agent formulations [12,13], and attention-augmented RUL-driven policies [19]—are formulated for homogeneous or purely discrete-state systems and do not natively support a continuous–discrete hybrid state, engineering action masking, or a data-quality objective. A faithful re-implementation in the present HS-MDP would therefore require substantial modification; the closest faithful representatives of their algorithmic cores (decentralized → S3, on-policy actor-critic → S4, value decomposition → S5) are already included.

### 6.3. Overall Performance Comparison

All four DRL methods were trained under the fairness protocol described in section above. Each method received the same final budget of [780,000] environment interactions and used the same set of independent training seeds.

In the simulation environment, the proposed method was trained for 3000 episodes, totaling approximately 7.8 × 10^5^ interaction steps. The average annual total cost and its subcategories across 30 random seeds for each strategy are summarized in Table 3. The results show that, from the engineering baseline perspective, periodic maintenance has the highest costs in all categories, while threshold-based CBM leverages state feedback to reduce costs by about 30.8% relative to periodic maintenance. From the DRL baseline perspective, Independent DQN further reduces costs by about 13.0% compared with threshold-based CBM, and PPO performs similarly to Vanilla-BDQ. The proposed method achieves a 60.3% cost reduction relative to periodic maintenance, a 42.6% reduction relative to threshold-based CBM, and a 34.2% reduction relative to Independent DQN. The largest contributors to the cost reduction are failure costs and data-quality loss, which decrease by 88.6% and 74.9%, respectively, reflecting a marked improvement in the avoidance of major risks.

**Figure 2:**
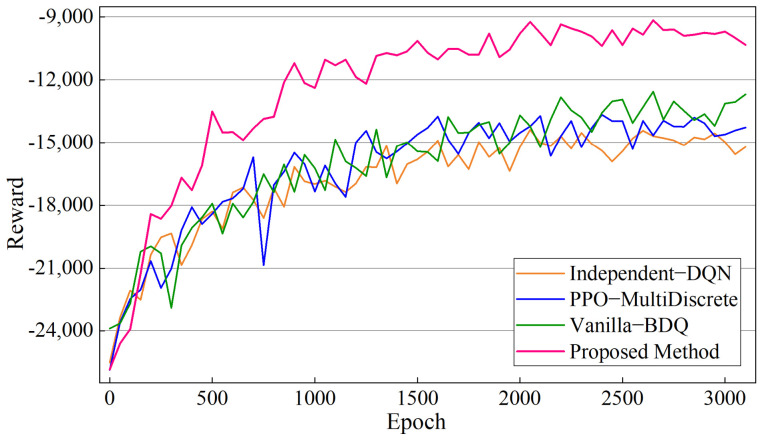
Comparison of episode reward curves during training.

In terms of reliability, the method proposed in this study yields a total of 1.4 failures per year across the four components, significantly lower than Periodic (9.8/year), Threshold-CBM (5.6/year), and Independent-DQN (3.7/year). In terms of operational availability, the method achieves 10.6 shutdowns per year, an annual cumulative downtime of 68.5 h (about 47% of Periodic), and a data efficiency of 96.2% (Periodic: 82.1%). The engineering significance lies in the markedly improved continuity of testing tasks—assuming the machine operates 16 h per day, each avoided unplanned failure prevents approximately 8–24 h of emergency downtime; compared with periodic maintenance, the method proposed in this study can recover about 52–156 h of effective operating time per year. Training curves and performance comparisons are shown in Figure 2.

The 60.3% reduction relative to periodic maintenance should be read together with its decomposition. The two categories that the fixed manufacturer schedule cannot react to—failure cost and data-quality loss—account for most of the gap, falling by 88.6% and 74.9%, respectively, whereas the maintenance and downtime costs, which periodic maintenance already incurs deliberately, decrease by a more moderate ~40%. The large headline figure therefore reflects the avoidance of high-consequence failures and out-of-tolerance data rather than a uniform across-the-board cost saving. Against the strongest learned baselines, the margin is correspondingly more modest but still consistent: the proposed method reduces total cost by 24.5% relative to Vanilla-BDQ and by 29.2% relative to PPO-MultiDiscrete, isolating the contribution of the inter-component attention and action masking from that of the BDQ backbone itself. All the experiment results has been listed in Table 4.

### 6.4. Opportunistic Maintenance and Cross-Component Attention Analysis

**Figure 3:**
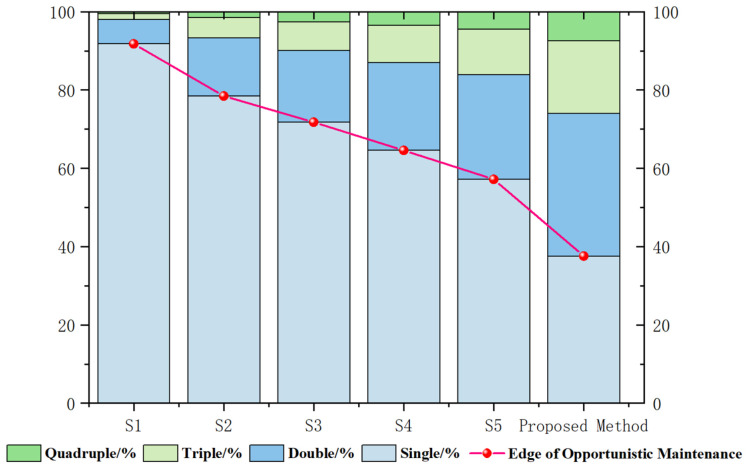
Comparison of opportunistic maintenance.

Define the opportunistic maintenance ratio ρ_opp as the proportion of “downtime events in which maintenance on multiple components is executed” to “the total number of downtime events.” The proposed method achieves ρ_opp = 62.4%, far higher than Threshold-CBM (21.5%) and Independent-DQN (28.2%). This proportion directly indicates that the proposed method actively captures the economic effect of “a single shutdown enabling parallel maintenance of multiple components”: among the 10.6 annual shutdowns, about 6.6 involve two or more components, and each opportunistic maintenance event involves an average of 2.5 components, as shown in Figure 3. Each part of this stacked bar chart represents maintenance downtime for a single component, two components, three components, and four components, respectively, and the red line represents the dividing line between single-component maintenance downtime and multi-component maintenance downtime.

**Figure 4:**
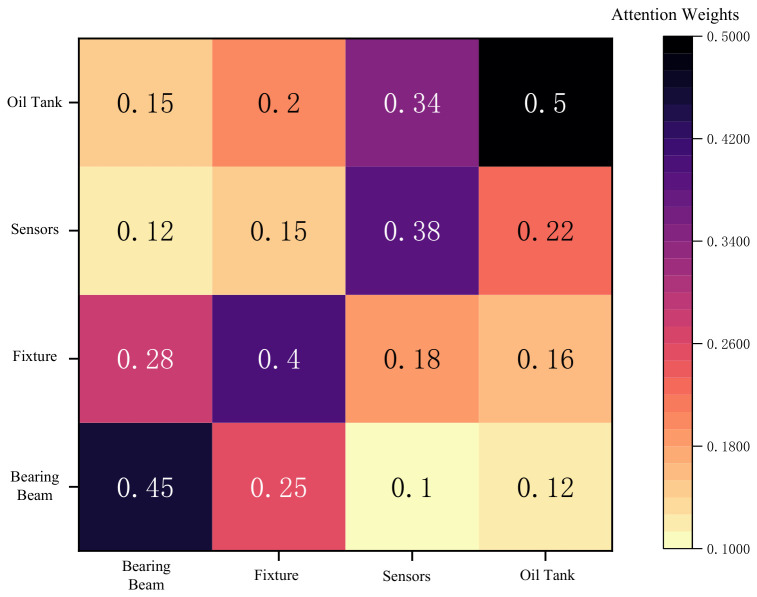
Heatmap of inter-component attention weights.

Furthermore, the inter-component attention weights of the trained policy network are visualized on the test set (Figure 4). The attention matrix exhibits clear off-diagonal elements: (1) Oil tank → sensors (α = 0.34): periods of elevated oil temperature and oil degradation typically coincide with high-load, high-frequency operating conditions, accelerating sensor degradation; (2) Fixture → bearing beam (α = 0.28): wear of the fixture leads to clamping misalignment, which imposes eccentric loading on the beam and accelerates horizontality degradation; (3) Sensors → oil tank (α = 0.22): sensor degradation can impair overload protection, indirectly causing oil overheating. These coupling patterns are highly consistent with the physical causal chain of the mechano-hydraulic system, demonstrating good interpretability of the inter-component attention mechanism.

### 6.5. Ablation Experiments

To quantitatively assess the independent contribution of each core component of the method, this study designs the following five ablation variants. All variants are retrained and evaluated in the same environment and with the same set of 30 random seeds, and the results are summarized in Table 5: (A) Full method (BDQ-Att-Mask); (B) −Attention: remove inter-component attention, with branching heads deciding directly based on shared encoder features; (C) −Mask: remove action masking, suppressing invalid actions via a reward penalty (−1000); (D) −Quality: remove the data-quality loss term from the reward function; (E) −Dueling: replace dueling heads with standard Q-heads.

Although Table 5 reports only the aggregate cost together with the annual failure and shutdown counts, these two counts already act as a decomposition lens: the failure count is a proxy for the failure-cost contribution and the shutdown count for the downtime contribution, so the source of each variant’s degradation can be inferred without additional instrumentation.

Removing action masking (C) is the most damaging variant (+30.9%) and degrades both proxies at once—failures rise from 1.4 to 3.6 per year and shutdowns from 10.6 to 13.8. Without the mask, feasibility and safety are enforced only through the −1000 reward penalty, which the agent must learn by repeatedly sampling infeasible actions; more importantly, the soft mask that forbids “no action” near the tolerance limit is lost, so critical maintenance windows are missed and the failure-driven cost dominates the increase.

Removing inter-component attention (B) raises the cost by 15.5% but adds only 0.6 shutdowns (10.6 → 11.2). The increase is therefore not explained by more frequent downtime but by poorer coordination within each downtime: the opportunistic–maintenance ratio falls from its full-method value of 62.4%, so maintenance that the complete model bundles into a single shutdown is now executed across separate visits, and the moderate rise in failures (to 2.2) reflects the loss of cross-component anticipation such as the oil-tank → sensor coupling.

Removing the data-quality term (D) is the most informative case. Its failure count (1.5) and shutdown count (10.4) are statistically indistinguishable from—indeed marginally lower than—those of the full method, yet the total cost rises by 18.0%. Because neither reliability nor availability has changed, the entire increase must originate in a cost dimension that these two columns do not capture, namely the silent loss of out-of-tolerance test data: once the term is removed, the agent’s incentive to maintain the sensors collapses, the frequency of the sensor reaching level 3 increases roughly 5.2-fold, and the policy pays through discarded data rather than through additional shutdowns. This is direct evidence that the data-quality term is not a redundant relabelling of existing costs but a distinct objective that reshapes the sensor-maintenance behaviour.

Removing the dueling decomposition (E) yields the smallest degradation (+7.1%), with failures and shutdowns close to the full method but a larger seed-to-seed standard deviation than the full method (820 vs. 702). Its contribution is therefore concentrated in optimization stability rather than in the structure of the converged policy.

### 6.6. Simulation-Based Robustness and Parameter Sensitivity Analysis

The following robustness and sensitivity analyses were performed entirely in the calibrated HS-MDP simulation environment. They were designed to examine the response of the learned policy to controlled model-parameter and cost-accounting perturbations. Unless otherwise stated, the policy trained under the nominal simulation parameters was frozen and evaluated without retraining.

For the degradation-model robustness test, the degradation-rate parameters in Table 3 were simultaneously perturbed by ±10%, ±20%, and ±30% in the evaluation environment. For each perturbation level, all compared policies were evaluated using the same 30 test seeds. This setting represents model misspecification between the nominal training simulator and the perturbed evaluation simulator.

(1) Degradation-Parameter Perturbation Robustness

**Figure 5:**
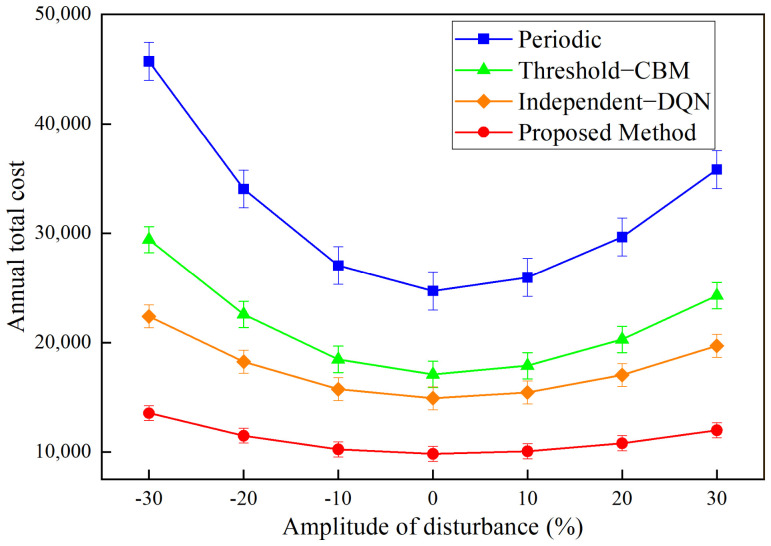
Robustness test.

In engineering practice, there is unavoidable uncertainty in estimating degradation parameters; therefore, it is necessary to assess the robustness of policy to parameter perturbations. We took three levels of relative perturbations (±10%,±20%,±30%) are simultaneously applied during evaluation. For each perturbation level, 30 random test seeds are generated to evaluate the average annual total cost of the method presented in this chapter (directly using the policy trained under baseline conditions, without retraining) and three baseline strategies. The results are shown in Figure 5.

The results indicate that when the degradation parameters are underestimated by 30%, the number of failures rises markedly for all strategies; the cost of the method presented in this chapter increases by about 38.2%, while Threshold-CBM increases by about 72.6%. When the degradation parameters are overestimated by 30%, all strategies show a stronger tendency toward over-maintenance; the cost of the method presented in this study increases by about 15.4%, and Periodic by about 21.8%. The method presented in this study exhibits the smallest performance degradation under both perturbation extremes, with robustness clearly surpassing all baselines. These results suggest that the DRL-learned policy does not “overfit” the degradation parameters used during training, but it instead has learned more generalizable state-based rules for multi-component coordinated decision-making.

(2) Cost-Parameter Sensitivity Analysis

Maintenance cost structures may vary significantly across enterprises and time periods. This section evaluates the transferability of the policy by perturbing cost parameters. Sensitivity is examined for three categories of cost parameters: the failure penalty coefficient (×0.5, ×1, ×2, ×4), the fixed downtime cost (×0.5, ×1, ×2, ×4), and the data-quality coefficient (×0.5, ×1, ×2, ×4). Under each perturbation level, the method presented in this chapter (using the policy trained under baseline conditions) is re-evaluated, and the results are summarized in Table 6.

The results show that the failure penalty is the most sensitive factor; however, even when amplified by 4×, the cost of the method proposed in this study increases by only +15.6%—because failures are already rare under the baseline policy (1.4 per year), so even when the per-failure cost is multiplied severalfold, its total contribution remains limited. For perturbations to the fixed downtime cost, the cost change is relatively larger (+22.8% at ×4) because this term directly couples with the number of shutdowns; nevertheless, the policy has learned to reduce shutdowns via opportunistic maintenance, limiting the amplification effect. The data-quality coefficient exhibits the lowest sensitivity, indicating that the sensor-maintenance policy has been trained into a robust behavior that is largely insensitive to this term. Taken together, these results indicate that the method proposed in this study maintains good transferability across different cost structures.

### 6.7. Discussion and Limitations

While the proposed BDQ-Att-Mask framework demonstrates substantial improvements in multi-component joint maintenance, it is fundamentally constrained by the assumption of full state observability. Currently, the HS-MDP relies on the accurate and continuous acquisition of structural degradation and discrete sensor states. Consequently, if sensor failures, missing data, or abnormal measurements occur in practical industrial environments, the decision-making agent will receive corrupted state inputs. This perturbation can lead to sub-optimal policies, such as missing critical opportunistic maintenance windows or executing redundant repairs. Furthermore, as the application extends to larger-scale industrial systems, the interactions between mechanical structures and hydraulic systems become significantly more complex. In such scenarios, relying solely on high-level macro-maintenance scheduling is insufficient to guarantee structural safety under severe state constraints or dynamic environmental disturbances. A highly promising future extension to address these intertwined limitations is transitioning towards a “Control-Maintenance Co-design” paradigm, which integrates deep reinforcement learning with advanced bottom-level robust control. Specifically, to mitigate the vulnerabilities associated with missing data and sensor anomalies, extended-state-observer (ESO) based adaptive control methods can be introduced as a state-estimation front end [25]. By utilizing ESOs to dynamically reconstruct the true system states under full-state constraints, the DRL agent can be supplied with filtered, robust state representations even when physical sensors fail. Additionally, incorporating optimized-based fault-tolerant control with disturbance rejection can ensure that the electro-hydraulic system maintains safe operational stability and rejects external disturbances while operating in a degraded state prior to the execution of scheduled maintenance [26].

## 7. Conclusions

Addressing the joint maintenance decision-making problem for multiple components in electro-hydraulic servo material fatigue testing equipment, this study proposes a method based on multi-head deep reinforcement learning. The main contributions and conclusions are as follows:

1. A continuous–discrete hybrid-state MDP modeling framework is constructed. The continuous degradation trajectories of the bearing beam and fixture are characterized by Wiener processes, while the main sensors and the hydraulic oil tank are modeled by discrete Markov chains to align with periodic calibration and oil-grade inspections in practice. A component-differentiated four-tier action space is designed, and engineering feasibility constraints are injected via action masking.
2. A multi-head Branching Dueling DQN network with an inter-component attention mechanism is proposed, reducing the parametric complexity of the joint action from ∏i|Ai| to  Σi|Ai|. Through a hierarchical design of shared encoder-attention layer- branching heads, component-wise decisions become aware of each other’s states and autonomously learn opportunistic maintenance patterns.
3. The reward function introduces a data-quality loss term determined by the sensor degradation level, aligning the optimization objective with the metrological attributes of the fatigue testing machine. Ablation experiments show that although this term appears to be merely one cost component, its removal leads to an overall relaxation of sensor maintenance and an 18.0% increase in implicit data-discarding costs.
4. The case study indicates that the proposed method reduces the average annual total cost by 60.3% relative to periodic maintenance and by 42.6% relative to threshold-based CBM; the number of failures drops from 9.8/year to 1.4/year, and data efficiency rises from 82.1% to 96.2%. The opportunistic maintenance ratio reaches 62.4%, far exceeding other baselines. Robustness tests and attention visualizations further validate the method’s transferability and interpretability.

Future work will focus on three directions: (1) transferring the policy trained in simulation to physical equipment and studying policy correction methods in Sim-to-Real settings; (2) extending to multi-machine collaborative maintenance scenarios, considering cross-machine spare parts sharing and the coupling with personnel scheduling; (3) exploring online adaptive estimation of degradation model parameters to further enhance the policy’s generalization to unknown operating conditions.

## Figures and Tables

**Table 1:** Definition of Discrete Degradation Levels for Sensors and the Hydraulic Oil Tank.

Component	Level	Meaning	Physical Correspondence
Sensor	1	Healthy	zero drift < 0.1% FS; sensitivity deviation < 0.2%
	2	Mild degradation	zero drift 0.1–0.3% FS
	3	Moderate degradation	zero drift 0.3–0.5% FS; affects data quality
	4	Failure	zero drift > 0.5% FS or sensitivity out of tolerance
Hydraulic Oil Tank	1	Excellent	≤NAS 6
	2	Normal	NAS 7–8
	3	Caution	NAS 9–10
	4	Severe contamination	NAS 11–12
	5	Replacement required	>NAS 12

**Table 2:** Component-Differentiated Action Space Design.

Component	Action 0	Action 1	Action 2	Action 3
Bearing Beam	No action (natural degradation)	Recheck and tighten (reduce subsequent drift rate)	$\mathrm{Leveling}\;\mathrm{adjustment}:\;h_{t+1}=(1-\rho_{1})h_{t}$	Full replacement: $h_{t+1}\;=\;0$
Fixture	No action	Clean and lubricate (slow subsequent wear)	$\mathrm{Grinding}\;\mathrm{repair}:\;w_{t+1}=(1-\rho_{2})w_{t}$	Full replacement: $w_{t+1}=0$
Sensors	No action	$\mathrm{Zero}\text{-}\mathrm{point}\;\mathrm{calibration}:\;z^{3}\;\to \;max(z^{3}-1,\;1)$	$\mathrm{Full}\;\mathrm{calibration}\;(\mathrm{reset}\;\mathrm{to}\;1\;\mathrm{if}\;z^{3}\;\leq \;3$)	$\mathrm{Replacement}:\;z^{3}=1$
Hydraulic Oil Tank	No action	Online filtration: $z^{4}\;\to \;max(z^{4}-1,\;1)$	Partial oil change: $z^{4}\;\to \;max(z^{4}-2,\;1)$	$\mathrm{Full}\;\mathrm{oil}\;\mathrm{change}:\;z^{4}=1$

**Table 3:** Parameter-provenance.

Quantity (Symbol)	Value	Source/Identification
Beam baseline drift $\mu_{h,0}$	1.2 × 10^−3^ mm/day	Fitted to 3 yr horizontality logs; cross-checked with maintenance manual
$\mathrm{Beam}\;\mathrm{failure}\;\mathrm{threshold}\;h_{\mathrm{crit}\;}$	0.50 mm	Alignment/horizontality tolerance for valid axial loading (cite governing standard, ISO 23788/ASTM E1012) [22]
$\mathrm{Beam}\;\mathrm{diffusion}\;\sigma_{h}$; coupling $p_{1},p_{2},\lambda$	$\sigma_{h}=5\times 10^{-4},p_{1}=2.0$ $p_{2}=1.2,\;\lambda =2.0$	Residual variance/regression on load- and frequency-stratified degradation rates
$\mathrm{Fixture}\;\mathrm{wear}\;\mathrm{rate}\;\mu_{w,0}$	4.5 × 10^−3^ mm/day	Fitted to clamping-wear logs
$\mathrm{Fixture}\;\mathrm{failure}\;\mathrm{threshold}\;w_{\mathrm{crit}\;}$; $\mathrm{diffusion}\;\sigma_{w}$	$w_{\mathrm{crit}\;}=0.8\;mm$ $\sigma_{w}=1\times 10^{-3}$	Clamping-slip tolerance from acceptance spec
Sensor level bands (zero drift 0.1/0.3/0.5% FS)	Table 1	Force/displacement calibration accuracy classes (ISO 7500-1 [23])
Oil contamination levels	NAS 1638 classes	NAS 1638 [24]
Base transition probs $p^{(3),0},p^{(4),0}$	$p^{(3),0}=0.004\;,0.006,\;0.008$ $p^{(4),0}=0.010,\;0.015,\;0.020,\;0.025$	Dwell-time MLE from work orders (formula above)
Acceleration coeffs β,α	β=0.05,α=0.07	Log-linear regression on frequency/oil temperature
$\mathrm{Load}\;\mathrm{amplitude}\;L_{t}$	~*U* [0.6, 1.4]	Usage/test-schedule logs
$\mathrm{Loading}\;\mathrm{frequency}\;f_{t}$	N within 10–30 Hz	Usage/test-schedule logs
Fixed downtime cost *C*_setup_; variable costs; failure penalty *c*_f_^(i)^	*C*_setup_ = 200 *c*_f_^(i)^ = 5000, 2000, 1500, 2000	3-yr corporate financial records (labor, materials, production-loss rate); scrapped-specimen cost
Data-quality coeffs *κ*_1_ < *κ*_2_ ≪ *κ*_3_	*κ*_1,_*κ*_2,_ *κ*_3_ = 20, 150, 1200	Cost of out-of-tolerance/discarded test data

**Table 4:** Comparison of average annual cost metrics for each strategy (unit: CNY).

Strategy	Maintenance Cost	Downtime Cost	Failure Cost	Data Quality Loss	Total Cost	Relative Periodic
S1 Periodic	8420 ± 210	5260 ± 180	6850 ± 920	4180 ± 310	24,710 ± 1220	—
S2 Threshold-CBM	6420 ± 228	4150 ± 172	3640 ± 712	2890 ± 245	17,100 ± 950	−30.8%
S3 Independent-DQN	5680 ± 305	4820 ± 256	2280 ± 520	2150 ± 228	14,930 ± 1085	−39.6%
S4 PPO-MultiDisc.	5950 ± 340	4150 ± 288	1950 ± 485	1820 ± 260	13,870 ± 1218	−43.9%
S5 Vanilla-BDQ	5520 ± 298	3980 ± 242	1820 ± 465	1690 ± 235	13,010 ± 1132	−47.4%
S6 BDQ-Att-Mask(Proposed Method)	4870 ± 185	3120 ± 162	780 ± 245	1050 ± 152	9820 ± 702	−60.3%

**Table 5:** Performance comparison of ablation experiments (mean over 30 seeds).

Variant	Total Cost(CNY)	Number of Failures	Number of Shutdowns	Relative Loss
A. BDQ-Att-Mask	9820 ± 702	1.4	10.6	—
B. −Attention	11,340 ± 905	2.2	11.2	+15.5%
C. −Mask	12,860 ± 1185	3.6	13.8	+30.9%
D. −Quality	11,580 ± 945	1.5	10.4	+18.0%
E. −Dueling	10,520 ± 820	1.7	11.0	+7.1%

**Table 6:** Cost-Parameter Sensitivity Analysis (relative cost changes with respect to the method proposed in this study).

Parameter	×0.5	×1.0 (Baseline)	×2.0	×4.0
Failure penalty coefficient	−18.5%	0	+8.2%	+15.6%
Fixed downtime cost	−12.4%	0	+10.5%	+22.8%
Data-quality coefficient	−8.6%	0	+5.2%	+12.4%

## Data Availability

The data that support the findings of this study are available upon reasonable request.
